# Etiology of Severe Childhood Pneumonia in The Gambia, West Africa, Determined by Conventional and Molecular Microbiological Analyses of Lung and Pleural Aspirate Samples

**DOI:** 10.1093/cid/ciu384

**Published:** 2014-05-27

**Authors:** Stephen R. C. Howie, Gerard A. J. Morris, Rafal Tokarz, Bernard E. Ebruke, Eunice M. Machuka, Readon C. Ideh, Osaretin Chimah, Ousman Secka, John Townend, Michel Dione, Claire Oluwalana, Malick Njie, Mariatou Jallow, Philip C. Hill, Martin Antonio, Brian Greenwood, Thomas Briese, Kim Mulholland, Tumani Corrah, W. Ian Lipkin, Richard A. Adegbola

**Affiliations:** 1 Medical Research Council Unit, Fajara, Republic of The Gambia; 2 Center for Infection and Immunity, Columbia University, New York, New York; 3 Ministry of Health and Social Welfare, Banjul, Republic of The Gambia; 4 Centre for International Health, University of Otago, Dunedin, New Zealand; 5 London School of Hygiene and Tropical Medicine, United Kingdom; 6 GlaxoSmithKline Vaccines, Wavre, Belgium

**Keywords:** etiology, pneumonia, children, Gambia, lung aspirate

## Abstract

Molecular analyses of lung aspirates from Gambian children with severe pneumonia detected pathogens more frequently than did culture and showed a predominance of bacteria, principally *Streptococcuspneumoniae*, >75% being of serotypes covered by current pneumococcal conjugate vaccines. Multiple pathogens were detected frequently, notably *Haemophilus influenzae* (mostly nontypeable) together with *S. pneumoniae*.

**(See the Editorial Commentary by Graham on pages 686–7.)**

Pneumonia remains the leading cause of death in children worldwide. A better understanding of the range of pneumonia pathogens is needed to reduce child mortality further [1], but this is hampered by limitations at the bedside and in the laboratory. Pneumonia etiologic studies have depended largely on blood culture, which is insensitive [2]. Direct percutaneous aspiration from the site of lung infection, rarely done despite its good safety record, yields the highest-quality clinical specimen, but even then bacterial culture identifies a pathogen in no more than half of cases [3].

Molecular methods have advantages over conventional methods for the detection and characterization of pathogens in clinical samples, and hold promise for improving diagnostic sensitivity [4]. We describe the application of both approaches to the detection of pathogens in lung and pleural aspirates obtained from children with severe pneumonia in a West African setting. The objective of the study was to elucidate the etiology of severe pneumonia in this group more comprehensively than has been possible previously.

## METHODS

The Gambia is a West African country of 1.8 million people with a human immunodeficiency virus (HIV) infection prevalence of <2%. A study of the etiology of severe childhood pneumonia was undertaken in the coastal area of The Gambia (Supplementary Data). At the time of the study (2007–2009), there was high coverage with conjugate *Haemophilus influenzae* type b (Hib) vaccine but no routine usage of pneumococcal conjugate vaccine (PCV), which is the case currently in many African countries. Cases were children aged 2–59 months with severe pneumonia defined clinically by modified World Health Organization (WHO) criteria (cough or difficulty in breathing, plus any of the following: lower chest wall indrawing, nasal flaring, or an oxygen saturation of <90% on pulse oximetry). Participants were recruited from the Medical Research Council (MRC) hospital in Fajara; the Royal Victoria Teaching Hospital in Banjul; and the major health centers at Fajikunda, Serekunda, and Brikama. Children with a cough of ≥2 weeks, or severe anemia (hemoglobin level <6 g/dL) or confirmed wheeze were excluded. Radiological pneumonia was defined using WHO criteria (ie, endpoint consolidation or pleural effusion). HIV testing was done if informed consent was given after standard counseling. A standard WHO guideline–based antibiotic regimen was used. Written informed consent was obtained for participation in the study from parents or guardians. The study was approved by the Gambian government/MRC Joint Ethics Committee (SCC/EC1062).

Details of lung aspiration in our setting over a period of 25 years, during which there have been no associated serious adverse events, have been described previously [5]. Participants underwent lung aspiration if they had accessible consolidation adjacent to the chest wall, had no contraindications, and written informed consent had been obtained. Pleural aspiration was undertaken in those with pleural effusions.

Culture, nonmolecular serotyping, HIV testing, singleplex polymerase chain reaction (PCR) for *lytA* and *cpsA* (for *Streptococcus pneumoniae*) and *glpQ* (for *H. influenzae*), 16S rRNA PCR, multilocus sequence typing (MLST), molecular serotyping, and multiplex fast-track 33 PCR, all using standard methods (Supplementary Data) [2, 6–13], were performed at the MRC Unit in The Gambia, and multiplex MassTag PCR [14] was performed at Columbia University. Multiplex pathogen targets are listed in Supplementary Data. Laboratory analyses were done blinded and independent of each other.

Summary results of organisms identified to genus or species level using 1 or more detection methods were compiled. Demographic and clinical data were double-entered into an SQL database (Microsoft Corporation) and verified, and laboratory data were entered into an Access (Microsoft Corporation) database, cross-checked, and verified. Statistical analyses were done using Stata software, version 11 (StataCorp).

## RESULTS

Fifty-five children, representing 74% of the radiological severe pneumonias and 26% of all clinical severe pneumonia cases identified in the study period, underwent lung aspiration (n = 47) or pleural fluid aspiration (n = 9); 1 participant underwent 2 aspirates at different time points (Supplementary Data). HIV testing was done in 33 of 55 (60%) participants, 2 of whom were positive for HIV type 1. The characteristics of those who underwent lung or pleural aspiration were similar to the radiological pneumonia group from which they were drawn (Supplementary Data).

Pathogens were identified to genus or species level in 53 of 56 (95%) samples by 1 or more laboratory methods (Table 1). An organism was cultured from 21 of 56 (38%) specimens: *S. pneumoniae* in 14 (25%), *H. influenzae* (all non–type b on latex agglutination) in 3 (5%), and *Staphylococcus aureus* in 3 (5%). Ziehl-Neelsen staining was done in 37 of 56 (66%) lung aspirate samples (all negative); 35 of 37 (95%) underwent culture for *Mycobacterium tuberculosis*, and all were negative.

**Table 1.** Organisms Identified to at Least Genus Level Detected in 53 Lung and Pleural Aspirate Specimens Examined by Both Culture and Molecular Assays Obtained From 52 Children With Severe Pneumonia

Organism	No.	(%)
Commonly recognized community-acquired pneumonia pathogens^a,b^		
*Streptococcus pneumoniae*	48	91
*Haemophilus influenzae*	12	23
*Staphylococcus aureus*	3	6
*Klebsiella* species	2	4
*Streptococcus* species (non-*pneumoniae*)	2	4
Bocavirus	2	4
Respiratory syncytial virus	2	4
Adenovirus	2	4
Enterovirus B	1	2
Coronavirus HKU1	1	2
Influenza C	1	2
Cytomegalovirus	1	2
Uncommonly reported community-acquired pneumonia pathogens		
*Acinetobacter* species^c^	3	6
*Enterobacter* species^c^	2	4
*Salmonella* species^d^	2	4
*Streptococcus pseudopneumoniae*^e^	1	2
*Bacteroides* species^f^	1	2
*Prevotella* species^f^	1	2
Codetection		
1 organism only	24	45
2 or more organisms	28	53
No organism	1	2
Bacterial organism(s) only	41	77
Viral organism(s) only	1	2
Bacterial–bacterial codetection	21	40
Bacterial–viral codetection	10	19
*S. pneumoniae* and *H. influenzae*	11	21
*S. pneumoniae* and *S. aureus*	1	2
*S. aureus* and *H. influenzae*	1	2

^a^ McIntosh K. Community-acquired pneumonia in children. N Engl J Med **2002**; 346:429–37.

^b^ Ruuskanen O, Lahti E, Jennings LC, Murdoch DR. Viral pneumonia. Lancet **2011**; 377:1264–75.

^c^ Jones RN. Microbial etiologies of hospital-acquired bacterial pneumonia and ventilator-associated bacterial pneumonia. Clin Infect Dis **2010**; 51(suppl 1):S81–7.

^d^ Genzen JR, Towle DM, Kravetz JD, Campbell SM. *Salmonella typhimurium* pulmonary infection in an immunocompetent patient. Conn Med **2008**; 72:139–42.

Mankhambo LA, Chiwaya KW, Phiri A, Graham SM. Lobar pneumonia caused by nontyphoidal *Salmonella* in a Malawian child. Pediatr Infect Dis **2006**; 25:1190–92.

^e^ Keith ER, Podmore RG, Anderson TP, Murdoch DR. Characteristics of *Streptococcus pseudopneumoniae* isolated from purulent sputum samples. J Clin Microbiol **2006**; 44:923–7.

^f^ Bartlett JG. Anaerobic bacterial infections of the lung and pleural space. Clin Infect Dis **1993**; 16(suppl 4):S248–55.

*Streptococcus pneumoniae* was detected by 1 or more molecular assays (Supplementary Data) in 48 of 53 (91%) samples and in 2 or more assays in 36 of 53 (68%) samples. Molecular pneumococcal serotyping (40 serotypes; Supplementary Data) showed the following prevalences: serotype 1, 22%; serotype 4, 18%; serotype 14, 18%; serotype 5, 16%; serotype 6A/B/C, 4%; and serotype 9V/A, 2%. Ten- and 13-valent PCVs both include serotypes that account for 76%–80% of serotypes identified in the samples in this study.

*Haemophilus influenzae* was detected in 12 of 53 (23%) samples. Four of the 12 had sufficient deoxyribonucleic acid (DNA) for full MLST typing, 3 being nontypeable *H. influenzae* (NTHi), whereas 1 in a child with HIV infection had a serotype b infection. Two of the 12 had sufficient loads for 5 or 6 of the standard 7 alleles of MLST to be defined, and these were suggestive of NTHi, 1 being in a culture-positive case confirmed as being non–type b by latex agglutination assay. *Staphylococcus aureus* was detected in 3 of 53 (6%) samples, all of which were pleural fluid specimens. One or more other bacteria were identified to genus or species level in 8 of 53 (15%) samples. Viruses, led by respiratory syncytial virus, adenovirus, and bocavirus, were detected by 1 or more methods in 10 of 53 (19%) samples.

Half (28/53 [53%]) of samples had >1 organism detected to genus or species level, predominantly 2 or more bacterial species. Codetection of *S. pneumoniae* and *H. influenzae* occurred in 11 of 53 (21%) samples, *H. influenzae* having the higher bacterial load in 4 of 11 samples. In the 3 specimens positive for *S. aureus*, low loads of *S. pneumoniae* were detected in 2 and of *H. influenzae* in the other.

## DISCUSSION

This study showed that *S. pneumoniae* was the predominant pathogen in this group of children with radiologic pneumonias, followed by *H. influenzae*, *S. aureus*, a range of gram-negative bacteria, and a few viruses. Potentially causative pathogens were found in all but 1 sample (98%), in contrast to the use of culture alone, which yielded an organism in 38% of specimens. *Streptococcus pneumoniae* and *H. influenzae,* predominantly NTHi, were detected together in around 1 in 5 cases in this Hib-vaccinated population. Current PCVs include >75% of serotypes identified in these samples.

Gambian studies of pneumonia etiology in the pre-Hib vaccine era showed a predominance of *S. pneumoniae* followed by Hib, as have other low-income country studies, including a recent lung aspirate study from Malawi using PCR [15, 16]. The role of NTHi in pneumonia has been raised before [17] and has been controversial [15].

It is likely that this study's findings are relevant to similar patient groups in other developing-country settings, especially those where conjugate Hib vaccine is routinely used and where PCVs have not yet been introduced. The multicountry Pneumonia Etiology Research for Child Health (PERCH) study will provide important data in this respect [4]. The prominence of *S. pneumoniae* but low frequency of Hib in our study appears to contrast with recent Global Burden of Disease 2010 estimates [18].

The strength of this study is its detailed analysis of the best possible specimens for diagnosing the cause of pneumonia using sensitive methods from a well-defined patient group that is representative of radiologic pneumonias. Multiple assays have provided several lines of supporting evidence, and 2 laboratories analyzed the raw clinical specimens, strengthening the findings. The predominance of *S. pneumoniae* along with its serotype distribution is supported by a high level of concordance between independent laboratories [19]. The study's weaknesses are that the best possible specimens are still subject to sampling error, that the study is relatively small, and that the identification of potential pathogens does not in itself confirm that these are the causative agents, a fundamental challenge for the field. Nevertheless, if there are any clinical samples for which pathogen detection alone is sufficient to assign causation, then these are lung and pleural aspirates.

The findings of this study emphasize the importance of bacteria, prominently *S. pneumoniae*, as a cause of severe pneumonia, and the potential for current PCVs to reduce the burden of this disease. This study also confirms that molecular methods are able to detect potential pathogens far more readily than culture and have a role in defining the etiology of pneumonia. The challenge of accurately assigning causation to the pathogens detected remains, particularly when using more generally available specimen types, and this will require the additional development of robust biomarkers of pathogen-specific disease.

## Supplementary Material

Supplementary Data
