# Supplementary material for: Etiology of Severe Childhood Pneumonia in The Gambia, West Africa, Determined by Conventional and Molecular Microbiological Analyses of Lung and Pleural Aspirate Samples
Source: Clin Infect Dis. 2014 May 27;59(5):682–5. doi: 10.1093/cid/ciu384 (PMC4130311; doi:10.1093/cid/ciu384)
Supplement: Supplementary Data [file ciu384_Supplementary_Data.zip › ciu384supp.docx]

**Appendix 1** Figure. Map of The Gambia showing the study area (hatched)

**MRC Hospital**

H


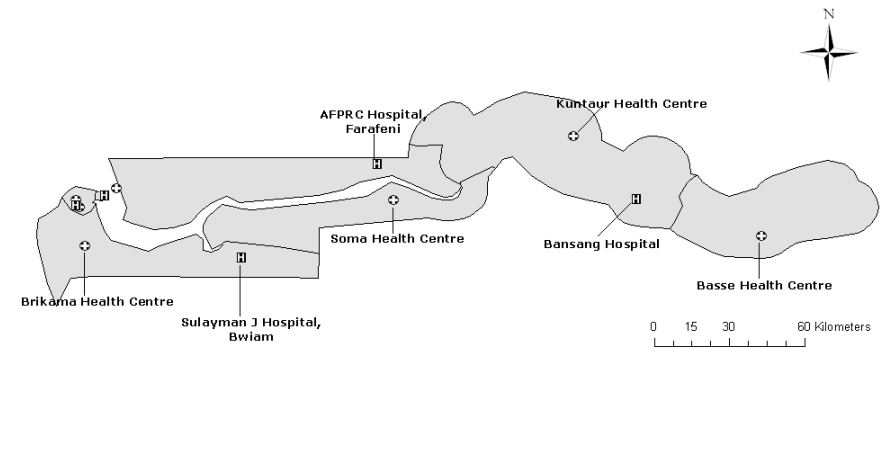

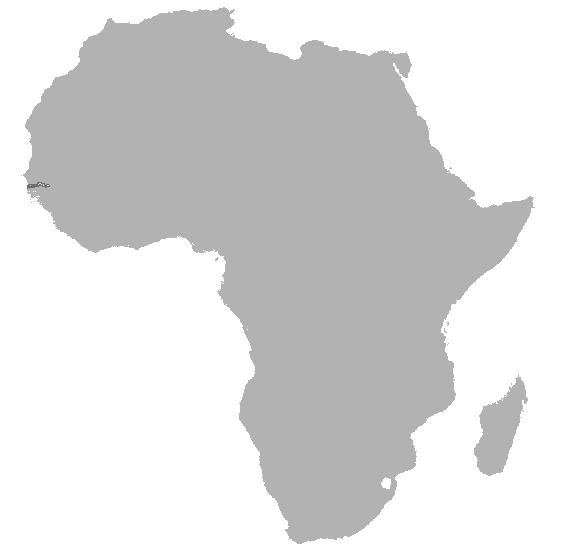

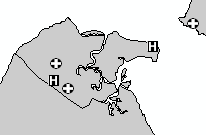


**The Gambia**

**Essau Health Centre**

**RVT Hospital**

**Serekunda Health Centre**

**Fajikunda Health Centre**

**JFP Hospital**

**Appendix 2.**

*Culture, non-molecular serotyping, and HIV testing*

Culture, non-molecular serotyping, and HIV testing were performed at the laboratories of the MRC Unit, The Gambia. Isolates of all bacteria were obtained by direct inoculation of culture media using standard microbiological procedures [[1](#_ENREF_1)]. Serotyping of *Haemophilus influenzae* isolates to determine if they were type b or non-type b was by latex agglutination (Directigen, BD). Serotyping of *Streptococcus pneumoniae* isolates was by latex agglutination and Quellung reaction [[2](#_ENREF_2)]. HIV testing was done by serology (Murex HIV1.2.0) and confirmatory HIV 1 and 2 PCR.

Specimens were subjected to gDNA extraction; *lytA, cpsA* and *glpQ* quantitative polymerase chain reaction (qPCR); *16S rRNA* PCR, qPCR and/or DNA sequencing; *S.pneumoniae-*specific and *H.influenzae-*specific multi-locus sequence typing (MLST); and molecular serotyping of *S.pneumoniae* isolates. Details of molecular targets, methods for DNA and RNA extraction, standard conditions for PCR, MLST, agarose gel electrophoresis, sequencing, bioinformatic applications and positive controls are given in this appendix. All primers were synthesized by Sigma Genosys (Texas) and were from previously published sources.

*Molecular analyses*

Singleplex PCR analyses, *16S rRNA*, MLST, molecular serotyping.

Singleplex PCR analyses (*lytA* and *cpsA* for *S. pneumoniae; glpQ* for *H. influenzae*), *16S rRNA* analyses, multi-locus sequence typing (MLST) and molecular serotyping were performed at the MRC Unit, The Gambia. Details are given in a previous publication [[3](#_ENREF_3)]. PCR results were defined as positive when amplification signals were observed on qPCR and specific size bands were observed on analysis of agarose gel electrophoresis of qPCR amplicons in the presence of an adequate amplification of the positive controls and absence of signal in the negative controls.

Multiplex analyses.

Specimens were subjected to multiplex MassTag PCR for bacteria and viruses at the laboratories of the Center for Infection and Immunity, University of Columbia, New York.[[4](#_ENREF_4), [5](#_ENREF_5)]. Nucleic acid was extracted from lung aspirate or pleural fluid using the easyMAG^®^ extraction platform (bioMériux, France). Samples were tested for 13 respiratory viruses and 7 respiratory bacteria, listed in Web Appendix 2. All MassTag PCR positive signals were confirmed by singleplex PCR and sequencing of PCR products.

Specimens also underwent separate nucleic acid extraction at the MRC Unit in The Gambia using the easyMAG^®^ platform and extracts were subjected to Fast-track multiplex PCR analyses (Fast-track Diagnostics, Luxembourg) for a panel of 21 respiratory viruses, listed in Web Appendix 2.

**Bacterial Pathogen molecular targets:** *S. pneumoniae* (*lytA, cpsA*) and *Haemophilus influenzae* (*glpQ*) [[6](#_ENREF_6), [7](#_ENREF_7)]; all bacteria including *Mycobacterium tuberculosis* (*16S rRNA)* [[8](#_ENREF_8), [9](#_ENREF_9)]; *Haemophilus influenzae* MLST *(adk, atpG, frdB, fucK, mdh, pgi, recA)* [[10](#_ENREF_10)]; *S. pneumoniae* MLST (*aroE, gdh, gki, recP, spi, xpt* and *ddl*) [[11](#_ENREF_11)]; molecular serotyping of pneumococcus using established targets [[7](#_ENREF_7)].

**Blinding and batching.** Samples were blinded on assignment of a laboratory number before being processed in batches for DNA extraction and qPCR.

**DNA extraction and *16S rRNA* standard PCR conditions**. DNA was extracted from a 200µl aliquot of each of the clinical samples using methods previously described [[3](#_ENREF_3)]. Extraction negative controls were included in each extraction batch to monitor for contamination in the system. The DNA eluate was stored at -20°C prior to qPCR. Each 25µl *16S rRNA* PCR and qPCR reaction was carried out as previously described [[3](#_ENREF_3)]. The positive control was a local isolate of *E. coli*. PCR results were defined as positive when amplification signals were observed on qPCR and specific size bands were observed on analysis of agarose gel electrophoresis of qPCR amplicons. DNA sequencing analysis was performed on a subset of PCR amplicons to ensure specificity of amplification and confirmation of bacterial species present. Samples were deemed negative if such a signal was absent or nonspecific bands were observed on qPCR product agarose gel electrophoresis, in the presence of an adequate amplification of the positive controls and absence of signal in the negative controls.

**MLST**. MLST was performed by preparing seven PCR amplicons from clinical sample extracts that were positive by qPCR for *S.pneumoniae* or *H.influenzae or both* as described previously for each specific organism [[10](#_ENREF_10), [11](#_ENREF_11)]. Matching of 7 alleles was required for full serotyping.

**Agarose gel electrophoresis**. All standard qPCR products and *S.pneumoniae* and *H.influenzae-*specific MLST PCR products were assessed for expected PCR product size on 2 % (w/v) agarose gels as previously described [[3](#_ENREF_3)].

**Nucleic acid sequencing**. PCR products were sent to Macrogen in South Korea ([http://www.macrogen.com](http://hinari-gw.who.int/whalecomwww.sciencedirect.com/whalecom0/science?_ob=RedirectURL&_method=externObjLink&_locator=url&_cdi=5188&_plusSign=%2B&_targetURL=http%253A%252F%252Fwww.macrogen.com%252F)) for purification and DNA sequencing.

**Bioinformatic applications**. Sequence analysis was performed as previously described [[3](#_ENREF_3)]. MLST alleles and sequence types were assigned to the isolate by comparing data to the *S.pneumoniae and., H.influenzae*  MLST database (http://spneumoniae.mlst.net/ & http://haemophilus.mlst.net/). Data for the newly identified STs were deposited in the *S.pneumoniae and., H.influenzae* MLST database <http://pubmlst.org//>. All resulting gene sequences from this study were deposited in Genbank.

**Positive and negative controls**. All positive controls were established through use of local isolates of bacteria by extracting and purifying nucleic acids and all gDNA and RNA extraction batches included extraction negative controls. All qPCR assays were run with extraction negative controls and non-template controls as well as positive controls and specificity controls.

Appendix 3.

**Pathogen targets for MassTag PCR**

influenza viruses A and B

human parainfluenza viruses 1-4

respiratory syncytial viruses (RSV) A and B

coronaviruses 229E and OC43

rhinoviruses

enteroviruses

adenovirus,

*Streptococcus pneumoniae*

*Chlamydophila pneumonia*

*Mycoplasma pneumonia*

*Mycobacterium tuberculosis*

*H. influenzae* type b

*Legionella pneumophila*

*Neisseria meningitides*

human metapneumovirus

**Pathogen targets for Fast-track PCR**

influenza viruses A, B and C

human parainfluenza viruses 1-4

respiratory syncytial viruses (RSV) A and B

coronaviruses 229E, OC43 NL63 (Cor63) and HKU1

rhinoviruses

enteroviruses

adenovirus

*Streptococcus pneumoniae*

*Chlamydophila pneumonia*

*Mycoplasma pneumonia*

*Mycobacterium tuberculosis*

*H. influenzae* type b

*Legionella pneumophila*

*Neisseria meningitides*

human metapneumoviruses A and B

parechovirus

bocavirus

cytomegalovirus

**Appendix 4 Figure** . Profile of participant inclusion in study and analysis^[[1]](#footnote-1)^

|  |  |  | | | Severe pneumonia cases recruited  N=207 | | | | | |  | |  | | |  | |
| --- | --- | --- | --- | --- | --- | --- | --- | --- | --- | --- | --- | --- | --- | --- | --- | --- | --- |
|  |  |  | | |  | | | | | |  | | No radiological pneumonia N=132 | | |  | |
|  |  |  | | | Cases with radiological pneumonia  N=75 | | | | | |  | |  | | |  | |
|  |  |  | | |  | | | | | |  | | Not eligible for aspiration N=18 | | |  | |
|  |  |  | | | Cases eligible for lung or pleural aspiration  N=57 | | | | | |  | |  | | |  | |
|  |  |  | | |  | | | | | |  | | Aspiration not done  N=2 | | |  | |
|  |  |  | | | Cases undergoing lung or pleural aspiration  N=55 | | | | | |  | |  | | |  | |
|  |  |  | | |  | | | | | |  | |  | | |  | |
|  |  |  | | | Samples collected  N=56 | | | | | |  | |  | | |  | |
|  |  |  | | |  | | | | | |  | | Unsuitable for molecular analysis N=1 | | |  | |
|  |  |  |  |  |  |  |  |  |  |  | |  | |  |  | |  |
|  | Culture  N=56 |  | *lytA*  N=53 |  | *cpsA*  N=48 |  | *glpQ*  N=53 |  | *16S rRNA*  N=53 |  | | MassTag  N=48 | |  | TB  Microscopy N=37  Culture N=35 | |  |

**Appendix 5**

**Table** . Characteristics of children with severe pneumonia who underwent lung or pleural aspiration (N=55), those of all children with radiological pneumonia (N=75) and all those with clinical severe pneumonia (undergoing or not undergoing aspiration) in the study group (N= 207).

* P-value for aspiration vs. radiological pneumonia, no aspiration

** P-value for aspiration vs. all other severes, no aspiration

Continuous variables were compared using Wilcoxon’s ranksum test

Categorical variables were compared using Fisher’s exact test

| Characteristic | | Aspiration group (N=55) | Radiological pneumonia group (N=75) | P-value* | All severe pneumonia cases (N=207) | P-value** |
| --- | --- | --- | --- | --- | --- | --- |
| Age in months (Median, (IQR)) | | 17 (12 to 29) | 17 (12 to 29) | 0.755 | 14 (7 to 25) | 0.002 |
| Gender (%) | Male | 56.4 | 56.0 | 1.000 | 54.6 | 0.875 |
|  | Female | 43.6 | 44.0 |  | 45.4 |  |
| Ethnicity (%) | Mandinka | 54.9 | 56.3 | 0.634 | 44.9 | 0.197 |
|  | Wollof | 17.7 | 14.1 |  | 13.4 |  |
|  | Fula | 11.8 | 11.3 |  | 15.5 |  |
|  | Jola | 7.8 | 8.4 |  | 11.3 |  |
|  | Other | 7.8 | 9.9 |  | 14.9 |  |
| Season of recruitment (%) | Rainy (June-Oct) | 32.7 | 36.0 | 0.416 | 47.3 | 0.012 |
|  | Dry (Nov-May) | 67.3 | 64.0 |  | 52.7 |  |
| Area of residence (%) | Banjul & Kanifing | 58.2 | 54.7 | 0.432 | 50.7 | 0.211 |
|  | Kombo Districts | 41.8 | 45.3 |  | 49.3 |  |
| HIV status (%) | Tested | 60.0 | 57.3 | 0.598 | 55.6 | 0.527 |
|  | Positive | 6.1 | 4.7 | 1.000 | 7.0 | 1.000 |
| Severity of pneumonia (%) | Severe | 87.3 | 88.0 | 1.000 | 89.4 | 0.611 |
|  | Very severe | 12.7 | 12.0 |  | 10.6 |  |
| Oxygen saturation (median, (IQR)) | | 96 (93 to 97) | 96 (94 to 97) | 0.699 | 96 (94 to 97) | 0.748 |
| White Cell Count x 10^9^ per litre (median, (IQR)) | | 23.2 (16.3 to 32.4) | 23.2 (16.3 to 32.6) | 0.875 | 16.1 (10.2 to 25.5) | <0.001 |
| Chest radiograph findings (%) | Significant pathology | 100.0 | 100.0 | 1.000 | 86.2 | <0.001 |
|  | Endpoint | 100.0 | 100.0 | 1.000 | 38.7 | <0.001 |

Appendix 6.

|  | *S. pneumoniae*  **N/D** (%) | *H. influenzae*  **N/D** (%) | *S. aureus*  **N/D** (%) |
| --- | --- | --- | --- |
| *Culture* | **14/56** (27) | **3/56** (5) | **3/56** (5) |
| *lytA* | **38/53** (72) |  |  |
| *cpsA* | **41/48** (85) |  |  |
| *glpQ* |  | **12/53** (23) |  |
| *16S rRNA* | **17/53** (32) | **6** **/53** (11) | **3/53** (6) |
| Mass Tag | **29/48** (60) | **2/48** (4) | **3/48** (6) |

**Table.** Detection of *S. pneumoniae,*  *H. influenzae* and *S. aureus* by culture and PCR assays (*lytA, cpsA, glpQ, 16SrRNA*, MassTag) in 56 lung aspirate (47) and pleural aspirate (9) samples. N=numerator, D=denominator

Appendix 7.

The *S. pneumoniae* serotypes included in molecular serotyping of samples are listed below.

| 14 |
| --- |
| 1 |
| 5 |
| 4 |
| 18A/B/C |
| 6A/B/C |
| 19F |
| 23F |
| 25F/38 |
| 9V/9A |
| 7C |
| 3 |
| 15B/C |
| 7F/A |
| 17F |
| 8 |
| 12F/A/44/46 |
| 9N/9L |
| 22F/A |
| 23A |
| 24A/B/F |
| 2 |
| 11A/D |
| 19A |
| 16F |
| 21 |
| 33F/A/37 |
| 15A/F |
| 35F/47 |
| 13 |
| 39 |
| 23B |
| 35A/C/42 |
| 20 |
| 35B |
| 10F/C |
| 34 |
| 10A |
| 31  6C/D |

Appendix 8. Organisms detected by 16SrRNA PCR but not able to be identified to at least genus level in 53 lung aspirate and pleural aspirate specimens obtained from 52 children with severe pneumonia

| **Organism** | N | % |
| --- | --- | --- |
| Gamma proteobacterium (not otherwise specified) | 8 | 15 |
| Moraxellaceae bacterium (not otherwise specified) | 3 | 6 |
| Coliform species (not otherwise specified) | 1 | 2 |
| Uncultured bacteria (not otherwise specified)^[[2]](#footnote-2)^ | 1 | 2 |

Appendix 9. Antibiotic sensitivities of *S. pneumoniae, H. influenza* and *S. aureus,* antibiotic treatment and outcome

*S. pneumoniae* isolates were sensitive to penicillin and chloramphenicol but resistant to cotrimoxazole, *H. influenzae* isolates were sensitive to chloramphenicol and 2 of 3 were sensitive to penicillin and ampicillin. There was no evidence of methicillin resistance amongst *S. aureus* isolates.

Nine children had their antibiotic treatment intensified *per protocol* because of deterioration or lack of improvement. One child died shortly after admission despite intensive treatment (with no evidence of pneumothorax), while all others recovered and were discharged.

Appendix 10. Pneumococcal serotyping and bacterial loads

Ten of 14 (71%) *S. pneumoniae* isolates were serotyped by conventional methods (Quellung) of which four were serotype 1, three serotype 5, two serotype 14, and one serotype 6B. Conventional and molecular serotyping concurred in 9/10 conventionally serotyped pneumococcal isolates, the one discrepancy being a case in which molecular serotyping identified serotype 14 while conventional serotyping identified serotype 5.

A range of bacterial loads was observed for *S. pneumoniae* (*lytA*) from 1 to >5x10^7^ genome copies per ml (median 2.7x10^4^, IQR 1.7x10^3^-4.3x10^5^), the highest loads being detected in pleural fluid samples. Bacterial loads for *H. influenzae* (*glpQ*) ranged from 17 to 5x10^5^ copies per ml (median 3x10^2^, IQR 6.7x10^1^-6.8x10^4^). Bacterial loads for *S. aureus,* identified in 3 empyema samples, were high measured semi-quantitatively by visualisation of bands on gel electrophoresis of *16S rRNA* qPCR products. The other organisms identified by *16S rRNA* were at low loads.

1. Adegbola, R.A., et al., *The etiology of pneumonia in malnourished and well-nourished Gambian children.* Pediatr Infect Dis J, 1994. **13**(11): p. 975-82.

2. Adegbola, R.A., et al., *Serotype and antimicrobial susceptibility patterns of isolates of Streptococcus pneumoniae causing invasive disease in The Gambia 1996-2003.* Trop Med Int Health, 2006. **11**(7): p. 1128-35.

3. Morris, G., et al., *A Novel Campylobacter jejuni Sequence Type from a Culture-Negative Patient in The Gambia.* PLoS ONE, 2008. **3**(3): p. e1773. doi:10.1371/journal.pone.0001773.

4. Briese, T., et al., *Diagnostic system for rapid and sensitive differential detection of pathogens.* Emerg Infect Dis, 2005. **11**(2): p. 310-3.

5. Tokarz, R., et al., *Longitudinal molecular microbial analysis of influenza-like illness in New York City, May 2009 through May 2010.* Virol J, 2011. **8**(1): p. 288.

6. Smith-Vaughan, H., et al., *Measuring nasal bacterial load and its association with otitis media.* BMC Ear Nose Throat Disord., 2006. **6**: p. 10.

7. Brito, D.A., M. Ramirez, and H. de Lencastre, *Serotyping Streptococcus pneumoniae by multiplex PCR.* J Clin Microbiol, 2003. **41**(6): p. 2378-84.

8. Chakravorty, S., et al., *A detailed analysis of 16S ribosomal RNA gene segments for the diagnosis of pathogenic bacteria.* J Microbiol Methods, 2007. **69**: p. 330-9.

9. Nadkarni, M., et al., *Determination of bacterial load by real-time PCR using a broad-range (universal) probe and primers set.* Microbiology, 2002. **148**: p. 257-266.

10. Meats, E., et al., *Characterization of encapsulated and noncapsulated Haemophilus influenzae and determination of phylogenetic relationships by multilocus sequence typing.* J Clin Microbiol, 2003. **41**(4): p. 1623-36.

11. Enright, M.C. and B.G. Spratt, *A multilocus sequence typing scheme for Streptococcus pneumoniae: identification of clones associated with serious invasive disease.* Microbiology, 1998. **144 ( Pt 11)**: p. 3049-60.

1. PCR targets listed are lytA and cpsA for *S. pneumoniae*, glpQ for H. influenzae, and 16S rRNA for all bacteria. MassTag is MassTag multiplex PCR [↑](#footnote-ref-1)
2. There were 4 instances where uncultured bacteria (not otherwise specified) were detected by *16S rRNA* PCR but only one of these where no other bacterium was identified by another means and this is the one that has been listed here. [↑](#footnote-ref-2)
